# Serum and tissue leptin in lung cancer: A meta-analysis

**DOI:** 10.18632/oncotarget.14963

**Published:** 2017-02-01

**Authors:** Xiang Tong, Yao Ma, Qilong Zhou, Jie He, Bo Peng, Sitong Liu, Zhipeng Yan, Xin Yang, Hong Fan

**Affiliations:** ^1^ Department of Respiratory and Critical Care Medicine, West China Hospital/West China School of Medicine, Sichuan University, Chengdu, 610041, China; ^2^ Innovative Drug Research Centre, Chongqing University, Chongqing, 404100, China; ^3^ Department of Respiratory Medicine, The First Affiliated Hospital of Chengdu Medical College, Chengdu, 610500, China; ^4^ Department of Internal Medicine, Hospital of Tradition Medicine LS.SC, Leshan, 614000, China

**Keywords:** lung cancer, cachexia, leptin, risk, meta-analysis

## Abstract

Many studies have found that leptin is involved in tumorigenesis and the progression of lung cancer. However, these studies were inconsistent. Therefore, we performed a meta-analysis to investigate the role of leptin in the patients with lung cancer. A systematic literature search in the several databases and on commercial Internet search engines was carried out to identify studies published up to July 8, 2016. The standardized mean difference (SMD) and odds ratio (OR) with 95% confidence interval (CI) were used to investigate the effect sizes. Finally, 21 eligible articles were included in the current meta-analysis. Overall, there is no relationship between levels of serum leptin and lung cancer. However, a subgroup analysis in high-study quality group found a weak association between serum leptin concentrations and lung cancer in Chinese (SMD=0.77, *P*=0.035). Additionally, the meta-analysis indicates that the serum leptin levels were lower in the weight-losing group than in the sustained weight group (SMD=-0.80, *P*=0.001). Further, there was evidence of a significant association between expression levels of leptin protein in tissue and lung cancer (OR=7.35, *P*<0.001). The present meta-analysis suggests that the serum and tissue leptin may be involved in the pathogenesis of lung cancer and tumor metastasis, especially among Chinese. However, the leptin may not appear to play an important role in cancer cachexia development.

## INTRODUCTION

Lung cancer is the most lethal malignancy. The most recent cancer statistics report estimated that lung cancer accounts for approximately 26% of all female cancer deaths and 29% of all male cancer deaths [1]. Despite several significant advances in cancer treatment, the 5-year relative survival for lung cancer is still less than 18% [1]. The main reasons contributing to the poor prognosis are the large population with advanced-stage lung cancer at diagnosis and the unsuccessful treatment in metastatic disease. Thus, there is intense interest in identifying novel biomarkers involved in this aggressive disease, for early diagnosis and to optimize its medical management. Previous studies have indicated many biomarkers considered as prognosticators, as well as indicators of screening and potential therapeutic targets for lung cancer, such as carcinoembryonic antigen, cytokeratin 19 fragment (CYFRA21-1), circulating cell free DNA, C-reactive protein, interleukin-6 (IL-6), and repetitive A (A type 3) [2–6].

Leptin, the product of the *ob* gene, is a cytokine synthesized and secreted mainly by adipocytes [7]. It inhibits food intake and regulates energy metabolism by inducing anorexigenic factors and suppressing orexigenic neuropeptides [8]. The published studies have suggested that food consumption is associated with a transient increase in serum leptin levels, whereas fasting reduces *ob* gene expression [7]. However, serum levels of leptin can be disturbed by other factors as well, such as various pro-inflammatory cytokines, including TNF-a, IL-1, and IL-6 [9]. Currently, leptin is also recognized as a polyfunctional cytokine, and it is considered to be involved in the pathogenesis of several cancer types, including breast cancer and pancreatic cancer [10–12]. Additionally, a growing body of studies has found that leptin is also involved in tumorigenesis and the progression of lung cancer. Among them, some studies have found that elevated serum leptin levels represent an independent risk factor for non-small-cell lung cancer (NSCLC) [13–15], whereas other studies have reported significant correlation between decreased serum leptin levels and prognosis in lung cancer [16, 17]. Further, a few studies have indicated that the expression of leptin protein was significantly higher in lung cancer tissues than in normal lung tissues [18, 19].

However, the observed relationships of these studies were inconsistent, and a single study may lack sufficient power to detect the possible small effect of the leptin levels on lung cancer, especially when the sample size is relatively small. Therefore, we carried out a most recent meta-analysis to accurately investigate the association between leptin and the risk of lung cancer, and to further assess the role of leptin in lung cancer.

## RESULTS

### Study characteristics

**Figure 1:**
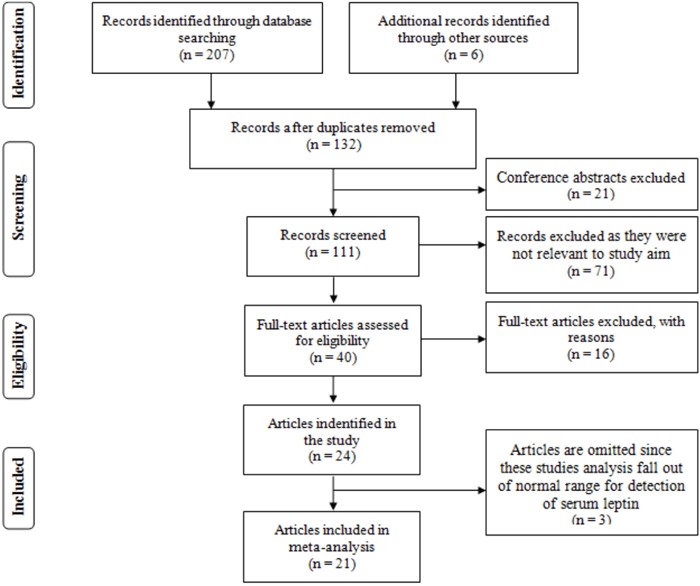
The flow diagram of the included and excluded studies

We initially searched in PubMed, Embase, the China National Knowledge Internet (CNKI), Wanfang databases, and commercial Internet search engines, and we identified 213 articles according to the search strategy. As showed in Figure 1, 81 studies were excluded because they were duplicated studies, and 21 items were excluded because they were conference abstracts. After title and abstract screening, 71 articles were removed because they were not relevant to our study aim. After further, full-view screening, nine articles were excluded because they were not relevant to lung cancer in relation to serum leptin concentrations and/or the leptin protein expression levels of tissue. Three articles were eliminated because they were reviews. Four articles were removed because they were possibly repeated studies. Therefore, 24 articles were identified [13–18, 20–37]. However, three articles are completely omitted because these studies analysis falls out of a normal range for detection of serum leptin [22, 27, 34]. Finally, 21 eligible articles were included in the current meta-analysis [13–18, 20, 21, 23–26, 28–33, 35–37].

Among them, fourteen included articles regarding the association between the serum leptin levels and lung cancer [13–17, 20, 24, 25, 28, 29, 31–33, 37], and seven included articles about the association between the leptin expression levels in tissue and lung cancer [18, 21, 23, 26, 30, 35, 36]. Ten of the 21 included articles were in English [13–18, 25, 29, 32, 33] and eleven were in Chinese [20, 21, 23, 24, 26, 28, 30, 31, 35–37]. Among the studies, fourteen articles were conducted in Chinese people [15, 18, 20, 21, 23, 24, 26, 28–31, 35–37], and seven studies were conducted in Europeans [13, 14, 16, 17, 25, 32, 33]. The characteristics of included studies are summarized in Tables 1, 2 and 3.

**Table 1:** Characteristics of studies involving association between the serum leptin and lung cancer

Author	Year	Ethnicity	Age	Type	Stage	Weight loss	
				NSCLC/SCLC	I/II/III/IV	n (%)	Status
Aleman MR	2002	Europeans	36-75/38-75	76/0	0/0/36/40	30 (23)	Untreated
Carpagnano GE	2007	Europeans	71±7/69±8	32/0	10/10/12/0	NR^1^	Untreated
Dong JH	2009	Chinese	47.32±10.01/34.46±5.61	NR	NR	NR	NR
Hong X	2013	Asians	51.61±10.41/46.69±10.46	0/41	NR	15 (36.6)	Untreated
Jamieson NB	2004	Europeans	43-79/46-74	20/0	NR	3 (16.5)	Untreated
Kerenidi T	2013	Europeans	62.9±9.2/NR	61/19	11/14/26/29	17 (21.3)	Untreated
Luo NP	2008	Chinese	53.0±12.0/50.0±15.0	NR	NR	NR	NR
Mou WJ	2014	Chinese	30-69/NR	27/0	0/0/4/21/2 (Unclear)	2 (7.4)	Untreated
Song CH	2014	Chinese	30-83//29-76	97/29	NR	NR	NR
Tao XN	2003	Chinese	32-71/29-68	25/9	0/17/13/4	NR	Untreated
Tas F	2005	Europeans	41-80/NR	28/0	NR	7 (25)	Untreated
Terzidis A	2009	Europeans	64.1±10.4/64.3±10.7	66/0	3/6/16/41	NR	NR
Werynska B	2009	Europeans	50-75/28-77	33/7	NR	20 (50)	Untreated
Zhang ZH	2003	Chinese	NR	NR	NR	NR	NR

^1^: Not report

**Table 2:** The levels of serum leptin in the each primary study

Author	Year	Cases			Controls			Unit	Method	Study Quality Score
		Mean	SD^1^	N	Mean	SD	N			
Aleman MR	2002	7.11	0.91	76	18.50	4.17	30	ng/ml	RIA^2^	7
Carpagnano GE	2007	45.3	19.9	32	22.7	0.4	20	ng/ml	ELISA^3^	7
Dong JH	2009	8.67	2.28	64	5.75	1.70	70	ng/ml	ELISA	7
Hong X	2013	6.45	2.55	41	7.05	2.61	51	ng/ml	ELISA	7
Jamieson NB	2004	6.7	11.85	20	15.8	9.1	13	ng/ml	ELISA	7
Kerenidi T	2013	3.87	8.80	80	10.76	8.42	40	ng/ml	RIA	7
Luo NP	2008	5.72	1.16	42	4.18	0.51	30	ng/ml	RIA	6
Mou WJ	2014	3.78	3.30	27	1.73	0.94	27	ng/ml	RIA	8
Song CH	2014	9.66	5.73	126	4.75	2.98	60	ng/ml	ELISA	7
Tao XN	2003	1.50	0.80	34	3.20	1.80	25	ng/ml	RIA	6
Tas F	2005	6.2	44.5	28	12.5	6.88	15	ng/ml	ELISA	5
Terzidis A	2009	9.30	9.20	66	7.1	5.8	132	ng/ml	RIA	7
Werynska B	2009	9.0	8.85	40	10	7.6	15	ng/ml	ELISA	6
Zhang ZH	2003	6.81	6.3	97	6.44	4.59	66	ng/ml	RIA	5
		Cases with Weight Loss			Cases without Weight Loss					
Hong X	2013	5.12	1.38	15	7.22	2.76	26	ng/ml	ELISA	7
Werynska B	2009	6.0	7.8	20	12	9	20	ng/ml	ELISA	6

^1^: standard deviation; 2: radioimmunoassay; 3: enzyme-linked immunosorbent assay

Table 3Characteristics of studies involving association between the tissue leptin expression and lung cancer

**Table:** a

Author	Year	Ethnicity	Age	Cancer/	Type	Stage	Case		Control		LN^3^ metastases (+/−)	Status
				Normal		I/II/III/IV	P^1^	N^2^	P	N		
Fang M	2008	Chinese	45-77	38/38	NSCLC^4^	6/15/17/0	27	11	10	28	27/11	Untreated
Guo SG	2010	Chinese	31-72	60/10	NSCLC	12/30/18/0	41	19	3	7	48/12	Untreated
Liu Y	2012	Chinese	59.8±8.6	68/68	NSCLC	18/25/25/0	49	19	18	50	NR^5^	Untreated
Sun LL	2011	Chinese	47-73	30/30	NSCLC	12/8/10/0	21	9	6	24	11/19	Untreated
Xu YJ	2011	Chinese	28-75	100/100	NSCLC	15/41/44/0	71	29	25	75	69/31	Untreated
Yang Y	2009	Chinese	57.8±5.7	40/40	NSCLC	15/17/8	34	6	16	24	25/15	Untreated
Zhang ZH	2010	Chinese	45-77	52/34	NSCLC	9/22/21/0	36	16	8	26	35/17	Untreated

**Table:** b

Author	Year	Method	LN metastases	Non-LN metastases	Ab^7^ types	Semi-quantitative score	Types of positive / negative controls
			Leptin (+/−)	Leptin (+/−)			
Fang M	2008	IHC^6^	21/6	6/5	Rabbit (donate)	>25%	adipose tissue / PBS^8^
Guo SG	2010	IHC	37/11	4/8	Rabbit (NR)	>20%	adipose tissue / PBS
Liu Y	2012	IHC	NR	NR	Goat ( Santa Cruz)	>25%	adipose tissue / PBS
Sun LL	2011	IHC	NR	NR	Rabbit (ZSGB-BIO)	>10%	adipose tissue / PBS
Xu YJ	2011	IHC	55/14	16/15	Rabbit ( Santa Cruz)	>25%	adipose tissue / PBS
Yang Y	2009	IHC	23/2	11/4	Rabbit (ZSGB-BIO)	>10%	adipose tissue / PBS
Zhang ZH	2010	IHC	27/8	9/8	Rabbit (ZSGB-BIO)	>25%	adipose tissue / PBS

1: positive; 2:negative; 3: lymph node; 4: non-small cell lung cancer; 5: not report; 6: immunological histological chemistry; 7: antibody; 8: phosphate-buffered saline

### Serum leptin

### Overall meta-analysis

**Figure 2:**
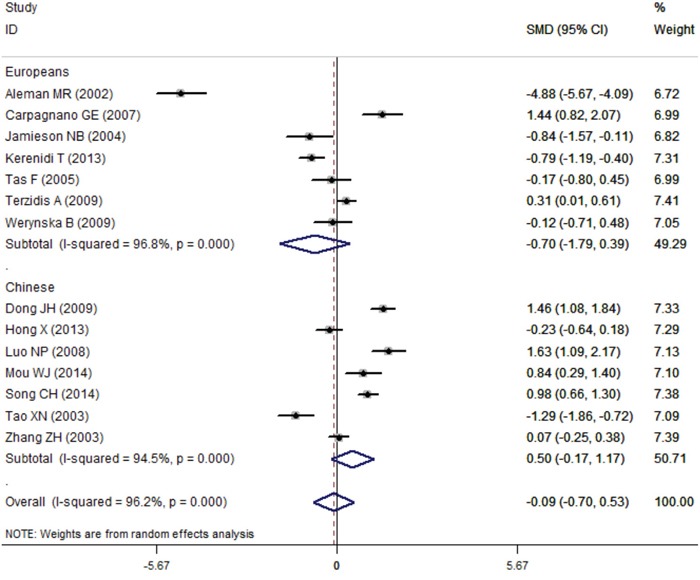
The results of association between the serum leptin levels and lung cancer Failure of this confidence interval to include zero indicates no statistical difference between the case group and control group. Negative SMD value suggests the mean of serum leptin concentration of case group is lower than that of control group, while positive SMD value suggest the mean of case group was higher than control group.

All 14 eligible case–control studies (773 cases and 594 controls) were included in the meta-analysis to investigate the association between serum leptin concentrations and lung cancer. As showed in Table 4, the overall meta-analysis results of the random-effect model suggested that no relationship exists between levels of serum leptin and lung cancer (SMD=-0.09, 95%CI=-0.70-0.53, *P*=0.780) (Figure 2). However, a non-ignorable heterogeneity among studies was found (*I*^2^=96.2%). Therefore, we further conducted subgroup analyses of different specific effects that were expected to lead to heterogeneity.

**Table 4:** The pooled results of the serum leptin levels in lung cancer patients compared with in health controls

	SMD^1^	95%CI^2^	P	I^2^ (%)^3^	Model
Overall	−0.09	−0.70-0.53	0.780	96.2	Random
Ethnicity					
Chinese	0.50	−0.17-1.17	0.144	94.5	Random
Europeans	−0.70	−1.79-0.39	0.208	96.8	Random
High quality group					
Overall	−0.16	−1.03-0.71	0.721	97.2	Random
Chinese	0.77	0.05-1.48	0.035	91.8	Random
Europeans	−0.93	−2.45-0.59	0.229	97.8	Random
Publish language					
In-English	−0.33	−1.21-0.54	0.456	96.6	Random
In-Chinese	0.33	−0.60-1.26	0.487	95.9	Random
Cancer types					
SCLC^4^	−0.23	−0.64-0.18	0.270	0	Fixed
NSCLC^5^	−0.53	−1.92-0.86	0.454	97.3	Random
Mixed	−0.30	−1.38-0.79	0.594	95.8	Random
NR^6^	1.04	−0.02-2.09	0.054	95.2	Random
Treatment status					
Untreated	−0.65	−1.57-0.26	0.159	95.9	Random
NR	0.87	0.30-1.44	0.003	92.3	Random

^1^: standardized mean difference; 2: confidence interval; 3: I-squared; 4: small cell lung cancer; 5: non-small cell lung cancer; 6: not report

### Subgroup meta-analysis

At first, we carried out a subgroup analysis by language (English or Chinese), but we found no statistical significance between serum leptin concentrations and lung cancer regardless of whether the studies were published in English or in Chinese (SMD=-0.33 95%CI=-1.21–0.54, *P*=0.456; SMD=0.33, 95%CI=-0.60–1.26, *P*=0.487, respectively). We further analyzed the results by the lung cancer types, and the results showed that the serum leptin levels were not statistically different between the NSCLC group and the SCLC group (NSCLC: SMD=-0.53, 95%CI=-1.92–0.86, *P*=0.454; SCLC: SMD=-0.23, 95%CI=-0.64–0.18, *P*=0.270).

**Figure 3:**
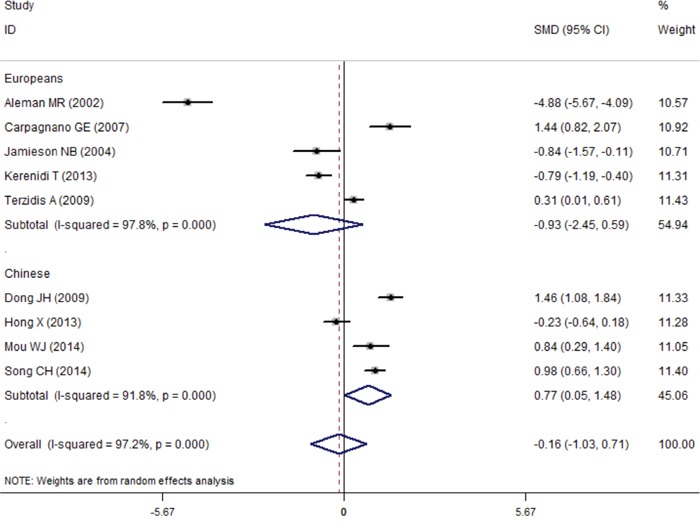
The subgroup analysis results of association between the serum leptin levels and lung cancer in high-study quality group (NOS score≥7) Failure of this confidence interval to include zero indicates no statistical difference between the case group and control group. Negative SMD value suggests the mean of serum leptin concentration of patients in high-study quality group is lower than that of control group, while positive SMD value suggest the mean of case group was higher than control group.

In the subgroup analysis of ethnicity, we found no association between serum leptin concentrations and lung cancer in Chinese people (SMD=0.50, 95%CI=-0.17–1.17, *P*=0.144) and in Europeans (SMD=-0.70, 95%CI=-1.79–0.39, *P*=0.208) (Figure 2). Interestingly, a weak statistical significance was found in the Chinese people of high-study quality group (NOS score≥7, 532cases and 443 controls) (Chinese: SMD=0.77, 95%CI=0.05–1.48, *P*=0.035; Europeans: SMD=-0.93, 95%CI=-2.45–0.59, *P*=0.229) (Figure 3). Additionally, the subgroup analysis of treatment status indicated there is no statistical significance between serum leptin concentrations and lung cancer in the non-treatment group (SMD=-0.65, 95%CI=-1.57–0.26, *P*=0.159), but there was a statistical association in the group that did not reported whether the patients received any treatment (SMD=0.87, 95%CI=0.30–1.44, *P*=0.003). All results of meta-analysis are summarized in Table 4.

### Sensitivity and meta-regression analysis

**Figure 4:**
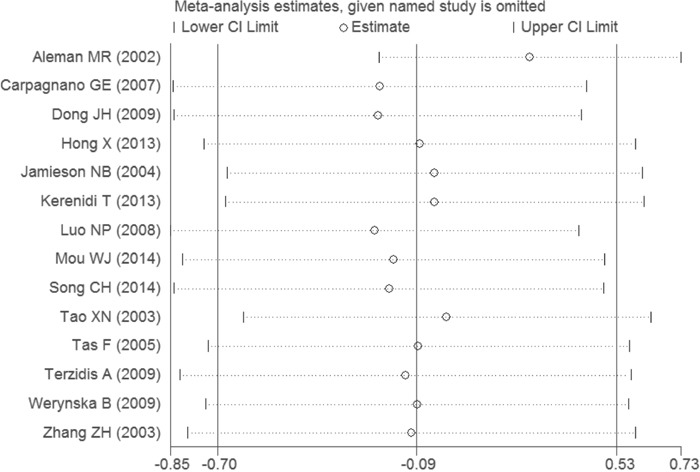
The result of sensitivity analysis on association between the serum leptin levels and lung cancer

**Figure 5:**
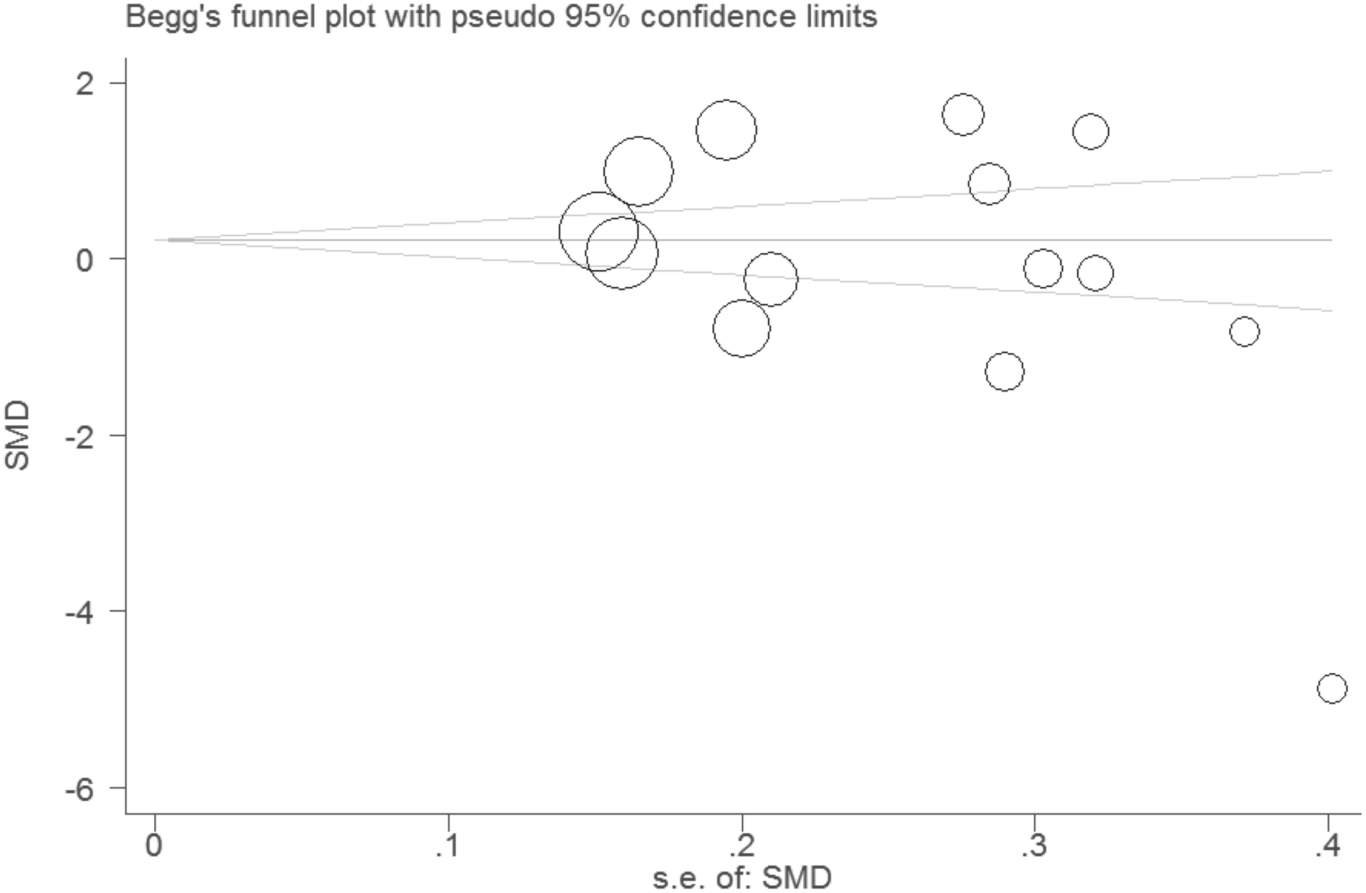
Funnel plot for evaluating publication bias on association between serum leptin levels and lung cancer Each circle represents a separate study for the indicated association. These circles in the funnel plot implied no asymmetrical distribution, which means no publication biases were found.

To further investigate the possible source of heterogeneity, we executed a sensitivity analysis by sequentially excluding studies from the meta-analysis to investigate the influence of each study on the pooled results. The result of sensitivity analysis found that the pooled ORs were not materially altered, suggesting the stability of our meta-analysis (Figure 4). Moreover, we conducted a multivariate meta-regression analysis to assess the possible confounding factors. The results of multivariate meta-regression analysis showed that the publish year, ethnicity, cancer type, and study quality as confounding factors did not substantially affect heterogeneity (adjusted *P* value is 0.079, 0.816, 0412 and 0.366, respectively). Further, no publication biases were found in the Begg's (*P*=0.101) and the Egger's tests (*P*=0.185) (Figure 5).

### Serum leptin in weight-losing group

**Figure 6:**
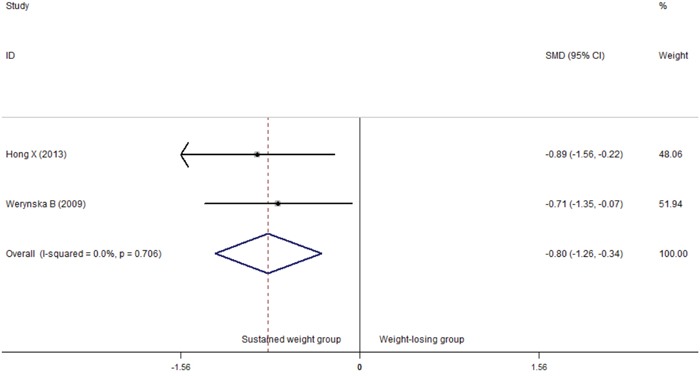
Comparison of differences of the serum leptin concentration in weight-losing group and in sustained weight group Failure of this confidence interval to include zero indicates no statistical difference between the weight-losing group and sustained weight group. Negative SMD value suggests the mean of serum leptin concentration of weight-losing group is lower than that of sustained weight group, while positive SMD value suggest the mean of weight-losing group was higher than sustained weight group.

Only two case–control studies (81 cases and 67 controls) compared the difference of serum leptin concentration in the weight-losing group with that in the non-weight-losing group. The results of the meta-analysis indicated that the serum leptin levels were lower in the weight-losing group than in the non-weight-losing group (SMD=-0.80, 95%CI=-1.26–(−0.34), *P*=0.001) (Figure 6).

### Tissue leptin

**Figure 7:**
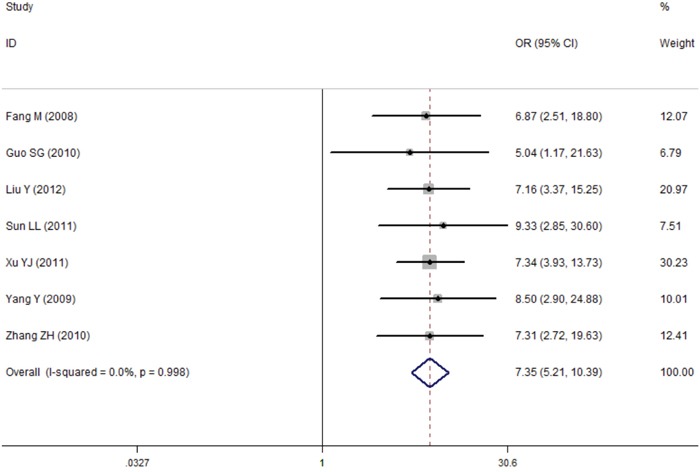
Comparison of difference of the leptin expression in lung cancer tissue and in normal tissue Failure of this confidence interval to include one indicates no statistical difference between the lung cancer tissue and normal tissue. Negative OR value suggests the mean of leptin expression of lung cancer tissue is lower than that of normal tissue, while positive OR value suggest the mean of lung cancer tissue was higher than normal tissue.

**Figure 8:**
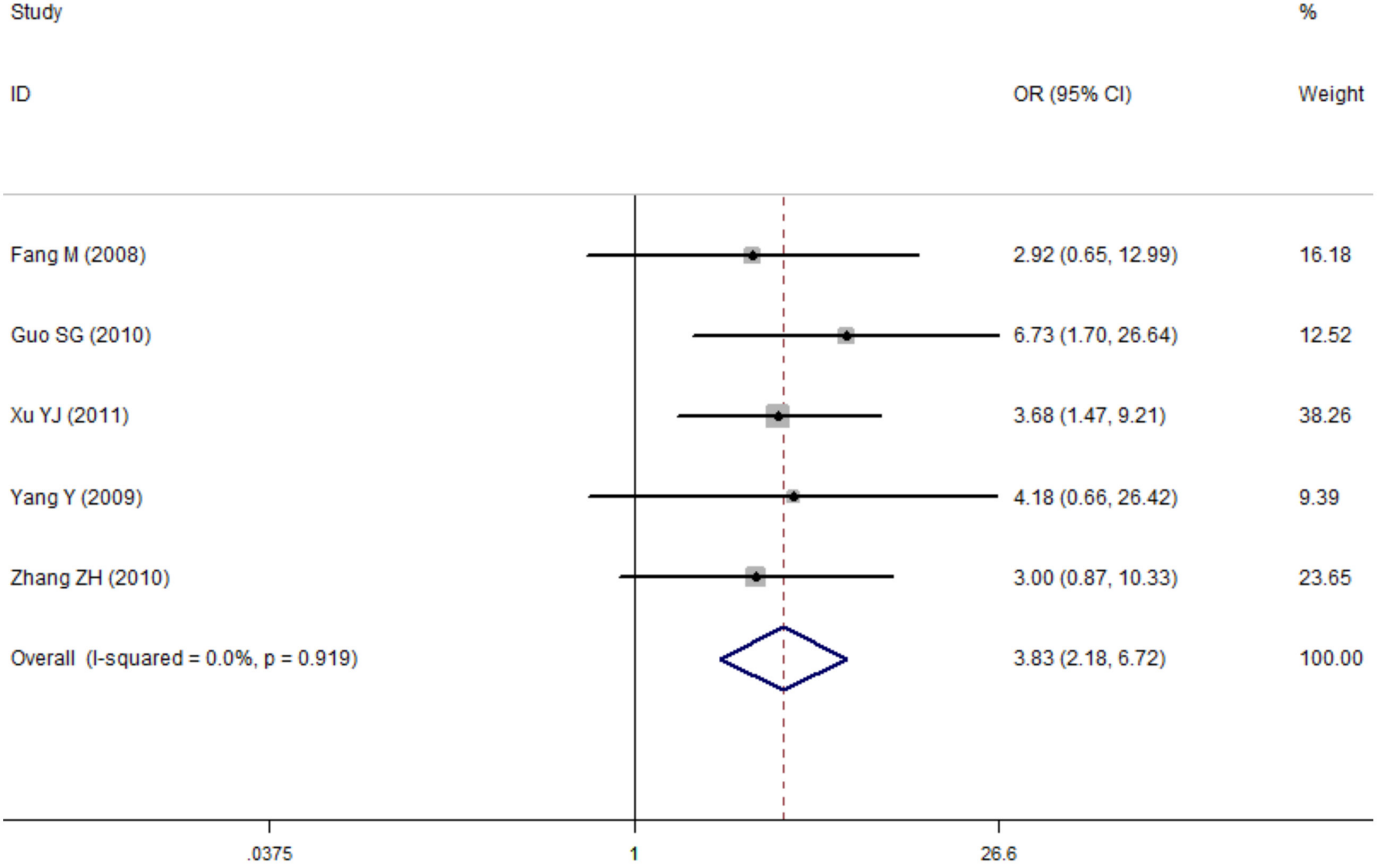
Comparison of difference of the leptin expression in the lymph node metastases group and the non-lymph node metastases group Failure of this confidence interval to include one indicates no statistical difference between the lymph node metastases group and the non-lymph node metastases group. Negative OR value suggests the mean of leptin expression of lymph node metastases tissue is lower than that of non-lymph node metastases tissue, while positive OR value suggest the mean of lymph node metastases tissue was higher than non-lymph node metastases tissue.

**Figure 9:**
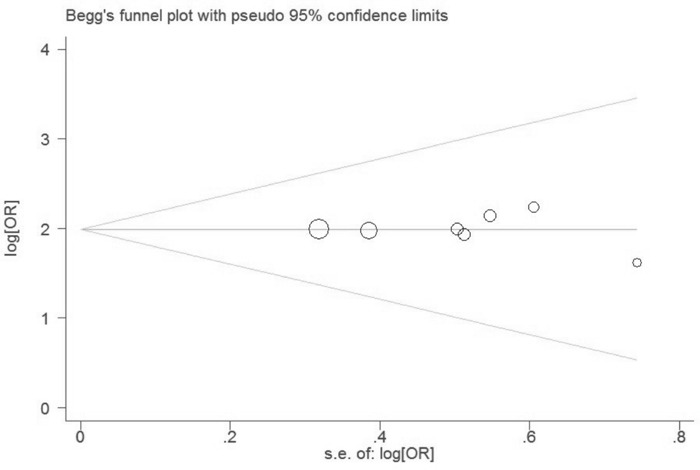
Funnel plot for evaluating publication bias on association between tissue leptin expression and lung cancer Each circle represents a separate study for the indicated association. These circles in the funnel plot implied no asymmetrical distribution, which means no publication biases were found.

In total, seven studies reported the role of leptin expression in lung cancer tissue, and all of these articles were conducted in China. For the meta-analysis, the fixed-effect model was used because there is no heterogeneity. The results indicated evidence for a significant association between expression levels of leptin protein in tissue and lung cancer (OR=7.35, 95%CI=5.21–10.39, *P*<0.001) (Figure 7). Interestingly, further analysis found that tissue leptin protein levels were statistically different between the lymph node metastases group and the non-lymph node metastases group (OR=3.83, 95%CI=2.18–6.72, *P*<0.001) (Figure 8). No publication bias was found in either the Begg's (*P*=1.0) or the Egger's test results (*P*=0.804) (Figure 9).

## DISCUSSION

Lung cancer is the most common cancer and the leading cause of cancer death in China [38, 39] (and worldwide [1]). It is well known that invasiveness and immortalization are important characteristics of cancer tissues, and postoperative recurrence and metastasis are the main reasons for ineffective treatment and death in patients with cancers. Therefore, the researchers are searching for specific biomarkers for early diagnosis of cancer or to distinguish the high risk of disease recurrence to achieve a better survival. Many studies have concluded that leptin may play a important role in the pathogenesis of lung cancer [13, 16, 30], but these results are inconsistent. Therefore, we undertook a meta-analysis to determine the value of serum and tissue leptin in lung cancer.

In the current meta-analysis, there were 14 primary studies comparing the blood leptin concentrations in lung cancer patients and healthy controls. The overall results indicated that the serum leptin levels did not increase in the patients with lung cancer, as compared with the controls. However, we found a non-ignorable heterogeneity (*I*^2^=96.2%) between studies in the meta-analysis. Although the heterogeneity can be taken into account by using the random-effect model, it would increase the probability of type-I error. The following factors may have contributed to the significant heterogeneity: (1) the demographic characteristics and genetic backgrounds are different in European and Chinese populations; (2) the studied patients with lung cancer had different stages of cancer in each study; (3) different lung cancer types were included in the studies; (4) there were different ratios of patients with weight loss in each primary study; (5) different measuring methods were used in the primary studies; (6) the included studies were of differing quality; and (7) the included patients had different treatment statuses.

To identify the causes of heterogeneity, at first, we carried out a sensitivity analysis by sequentially excluding each study. Fortunately, the statistically similar results were obtained, suggesting the stability of the meta-analysis. Second, we also conducted a multivariate meta-regression analysis to further assess the possible confounding factors. However, the results suggested that the publish year, ethnicity, cancer type, and study quality, as the confounding factors, did not substantially affect the heterogeneity. Therefore, we finally conducted subgroup analyses of different specific effects.

The serum leptin levels still did not differ between the patients with lung cancer and the healthy participants in the subgroups analyses (lung cancer types, published language, and treatment status). However, the subgroup analysis of ethnicity in the high-study quality group (NOS score≥7) indicated that the Chinese patients with lung cancer had higher serum leptin levels than the healthy population. Interestingly, our meta-analysis results were consistent with previous studies reporting that patients with hepatocellular carcinoma or prostate cancer have higher leptin levels than the normal population does [40, 41].

In addition, high expression levels of tumor-associated leptin receptor are thought to promote tumor growth and progression [42]. Therefore, we also carried out a meta-analysis to investigate the role of tissue leptin protein expression in lung cancer. The results indicated that the positive expression of leptin protein is obviously higher in lung cancer tissue than in normal tissue, especially significantly higher in the lymph node metastases group. Surprisingly, an earlier study also suggested that the increased detection of *ob*-R in ovarian cancers was associated with decreased survival [43]. In animal experiments, deficiency of the leptin receptor resulted in failure of mammary tumor formation [44]. The integrin-dependent migration of chondrosarcoma cells by involvement of IRS-1/PI3K-dependent activation of Akt was canceled with the knockdown of the long form of the leptin receptor [45]. Thus, even though the mechanism remains completely unclear, we speculate that leptin may play an important role in the pathogenesis of lung cancer and tumor metastasis, and the increased serum leptin level may possibly predict the diagnosis and progression of lung cancer. Further, the results of previous studies and the current study may help us to identify a new molecular marker for lung cancer diagnosis and a target for treatment.

Moreover, cancer anorexia-cachexia syndrome causes health deterioration and is a negative predictive factor of treatment response [33]. In addition, a previous study has shown that leptin is a protein hormone that functions as the afferent signal in a negative feedback loop regulating body mass [46]. Therefore, we analyzed the role of serum leptin in lung cancer patients with weight loss. Interestingly, the results suggested that serum leptin levels were lower in the weight-losing group than in the patients without weight loss and in the normal participants. Similar results were found in Weryńska's [33] and Jamieson's [25] studies. These results and our meta-analysis results indicate that changes of serum leptin levels should be considered to be the result of cachexia and not the cause of it, because its concentration depends on the total body fat mass. However, because only a small number of included studies refer to these results, the evidence is relatively weak.

There were several limitations of this meta-analysis. First, even if no publication bias was observed using several tests, published studies were identified in only a few databases. Therefore, there may be other biases in the present study. Second, because sufficient data in primary studies are lacking, we failed to perform further subgroup analyses to investigate the other factors, such as gender, age, and cancer stage, which may have affected our results. Third, the subjects of this meta-analysis mainly came from China, so the results are possibly only applicable to the Chinese. Despite these limitations, we minimized the likelihood of bias through the whole process by creating a detailed protocol and by performing study identification, data selection, and statistical analysis, and we controlled for publication bias.

In summary, the current meta-analysis suggests that the serum and tissue leptin may play an important role in the pathogenesis of lung cancer and tumor metastasis, especially among Chinese. However, leptin may not be involved in cancer cachexia development. We recommend that researchers design more rigorous and uniform case–control or cohort studies to confirm the results in the future.

## MATERIALS AND METHODS

### Literature search

We performed a systematic literature search in the PubMed, Embase, Wanfang databases, and CNKI, to identify studies involving the role of leptin in lung cancer up to July 8, 2016. The key search terms were as follows: *lung cancer, non-small-cell lung cancer, small-cell lung cancer, NSCLC, SCLC*, and *leptin*. The meta-analysis was limited to studies published in English or Chinese. Additionally, we also conducted a web-based search using many commercial Internet search engines (such as Google and Baidu), using the same keywords. Further, the reference lists of the obtained articles were also reviewed.

### Study selection

The inclusive criteria were as follows: (1) a study involving the role of serum leptin levels in lung cancer designed as a case–control study; (2) a study involving expression levels of leptin in tissue comparing lung cancer tissues with the normal tissues; (3) a primary study providing available data for calculating standardized mean difference (SMD) or odds ratio (OR) with 95% confidence interval (CI); and (4) the subjects of the study are human. The exclusion criteria were as follows: (1) lack of control cohort; (2) review and overlapping study; and (3) the study does not show the available data and is missing other essential information.

### Study quality score evaluation

The qualities of included studies involving the role of serum leptin levels in lung cancer were assessed by using the Newcastle–Ottawa Scale (NOS) for case–control study, to investigate quality based on three aspects: selection, comparability, and exposure in the primary study. The total score ranged from 0 to 9 (0–3, 4–6, and 7–9 was considered low, moderate, and high quality, respectively).

### Data extraction

Two independent authors (Xiang Tong and Yao Ma) collected the detail information and data from each study and used a predesigned data extraction Excel form. If there was any disagreement, the third author (Qilong Zhou) would further assess these articles. The information and data were extracted as follows: first author, publication year, country, ethnicity, sample size, age of participant, lung cancer type, stage of cancer, rate of weight loss, serum leptin levels (mean and standard deviation), leptin expression levels in tissue, treatment status, and test method.

### Statistical methods

In the present study, the SMD with 95% CI was applied to compare the serum levels of leptin in the patients with lung cancer with the levels in healthy controls, whereas the OR and 95% CI were used to investigate the leptin expression levels in lung cancer tissue and in normal tissue. We calculated the heterogeneity by the χ^2^ based Q-test and *I*-squared (*I*^2^) statistics test. The pooled effect size (SMD and OR) would be assessed using a random-effect model if heterogeneity was considered statistically significant (*I*^2^ > 50% and *P* < 0.10); if not, the fixed-effect model was used. To determinate the ethnicity, lung cancer type, and study quality-specific effects, we also performed subgroup analyses of different specific effects.

In addition, publication bias was assessed using several methods [47, 48]. The Begg's and Egger's tests were used to assess publication bias. The visual inspection of asymmetry in funnel plots was carried out to further investigate the publication bias. All data analyses were conducted using the STATA 12.0 software.
